# The effectiveness of full and partial travel bans against COVID-19 spread in Australia for travellers from China during and after the epidemic peak in China

**DOI:** 10.1093/jtm/taaa081

**Published:** 2020-05-22

**Authors:** Valentina Costantino, David J Heslop, C Raina MacIntyre

**Affiliations:** 1 The Biosecurity Program, The Kirby Institute, UNSW Medicine, The University of New South Wales, Australia; 2 The School of Public Health and Community Medicine, UNSW Medicine, The University of New South Wales, Australia

**Keywords:** pandemic, outbreak response, no-pharmaceutical interventions, modelling, boarder closure, epidemic control, coronavirus

## Abstract

**Background:**

Australia implemented a travel ban on China on February 1^st^ 2020, while COVID-19 was largely localised to China. We modelled three scenarios to test the impact of travel bans on epidemic control. Scenario one was no ban, scenario two and three were the current ban followed by a full or partial lifting (allow over 100 000 university students to enter Australia, but not tourists) from the 8^th^ of March 2020.

**Methods:**

We used disease incidence data from China and air travel passenger movements between China and Australia during and after the epidemic peak in China, derived from incoming passenger arrival cards. We used the estimated incidence of disease in China, using data on expected proportion of under-ascertainment of cases, and an age specific deterministic model to model the epidemic in each scenario.

**Results:**

The modelled epidemic with the full ban fitted the observed incidence of cases well, predicting 57 cases on March 6^th^ in Australia, compared to 66 observed on this date, however we did not account for imported cases from other countries. The modelled impact without a travel ban results in more than 2000 cases and about 400 deaths, if the epidemic remained localised to China and no importations from other countries occurred. The full travel ban reduced cases by about 86%, while the impact of a partial lifting of the ban is minimal, and may be a policy option.

**Conclusions:**

Travel restrictions were highly effective for containing the COVID-19 epidemic in Australia during the epidemic peak in China and averted a much larger epidemic at a time when COVID-19 was largely localised to China. This research demonstrates the effectiveness of travel bans applied to countries with high disease incidence. This research can inform decisions on placing or lifting travel bans as a control measure for the COVID-19 epidemic.

**Introduction**

In response to the epidemic of COVID-19, [1] Australia implemented a travel ban from China on February 1st 2020, adding Iran and then South Korea and Italy to the ban on February 29th, March 5th and 10th respectively. In addition, Australians evacuated from Wuhan and from the Diamond Princess cruise ship were quarantined for two weeks in dedicated quarantine facilities. The ban on travel from China has been periodically reviewed, with lifting of restrictions announced on February 23rd for high school students, who number less than 800. In contrast, over 120000 university students are unable to enter Australia to commence or resume their studies, and a booming tourism industry has ceased.

Non-pharmaceutical interventions measures like social distancing and quarantine are effective public health tools to control epidemic diseases [2], and Australia successfully delayed the introduction of the 1918 influenza A H1N1 pandemic by 1 year and reduced the total mortality compared to other countries [3]. However, evidence on the effectiveness of travel bans in containing the global spread of emerging infectious diseases is still limited [4] and they are not sustainable indefinitely. Therefore, a careful risk analysis needs to be done comparing the health and economic consequences implementing travel bans for the control of COVID-19 for alternative scenarios of increasing and decreasing disease incidence. The epidemic in China peaked on February 5th and has declined since [5]. The risk of importation of COVID-19 cases, firstly documented by the Bluedot group in Canada [6,7], through travel from an affected country is proportional to the volume of travel from that country and their prevalence of infection at that time point.

We aimed to estimate the impact of the implementation of the travel ban on China from February 1st, 2020 on the epidemic trajectory in Australia, as well as the impact of lifting the ban completely or partially from the 8 of March, when disease incidence was waning in China.

**Methods:**

Three scenarios were considered.

1. No travel ban – the epidemic curve if the travel ban was never placed
2. Complete travel ban from February 1st to March 8th, followed by complete lifting ban
3. Complete travel ban from February 1st to March 8th, followed by partial lifting ban (allowing university students, but not tourists, to enter the country)

***Estimation of infected cases coming into Australia from China***

The evacuations from Wuhan and the Diamond Princess Cruise ship to Australia are not considered in this model, which only examines regular air travel between China and Australia. In order to estimate the effectiveness of the travel ban that has been implemented in Australia for travellers from China, we did not consider bans to other countries. We assumed that the chance of cases coming into Australia from China depends from the number of cases in China and the number of travellers to Australia. To estimate the number of people infected that are predicted to enter Australia every two weeks from 20/01 to April, we utilised 2019 air travel passenger movements between China and Australia, derived from incoming passenger arrival cards, with data aggregated monthly and published by the Australia Bureau of Statistics (ABS) [8]. ABS incoming passenger card statistics are disaggregated based on permanent resident or citizen returning, permanent migration, short term stay and long-term arrivals. Long term arrivals are defined as > 1 year. For the purpose of this analysis air movements of passengers between China and Australia were derived from 2019 data. A baseline level of entries into Australia from China was calculated from the total number of entries over the April – Jun 2019 time period and was assumed to represent the baseline arrivals for the purpose of tourism and other business. The seasonal excess of travellers was then calculated by deducting this baseline from the January-March 2019 data. The seasonal excess arrivals were assumed to represent the arrival of international students starting the 2019 study year, which begins in February to March each year. Where travel bans were instituted in this analysis, or lifted, it was assumed that international students unable to enter Australia would return to Australia following the lifting of the ban, 60% in the rest of March and the remaining 40% over the month of April. However, tourists not able to travel during a travel ban were not assumed to enter Australia at a later date. Tourism activity was assumed to recover to baseline levels immediately after the lifting of a travel ban. We assumed that after lifting the ban, all the students that could not get in during ban will enter in addition to the new ones, however tourists that did not enter Australia for the ban are not assumed to enter when the ban is lifted. Once the ban is lifted, partially or not, we consider the situation to be normal again, due to not having enough information to base different future assumptions on. It is possible that after lifting of the ban, travel will be reduced from past baseline numbers, which would reduce the risk of importations even more. The daily number of travellers from China to Australia in each month and for each scenario is showed in Table S2 of the supplementary material.

To then calculate the probable number of those that could be infected we used an epidemiological dataset of confirmed cases of COVID-19 in China collected from WHO situation reports [9] and available in our supplementary materials (Table S1). The dataset includes all confirmed cases in China reported from 31/12/2019 to 23/02/2020. We then assumed that notified cases reflect only 10% of the real new infections per day, due to under-reporting, mild cases and asymptomatic infections. This assumption is based on data from Japan [10], which estimated that only 9.2% of cases in China were notified or detected. This estimate is based on testing of all evacuees from Wuhan to Japan and the documented cases in China at the time [10]. Furthermore it has been showed that a high proportion of infected people will have very mild symptoms [11] which are unlikely to be reported. We then estimated the possible true epidemic curve. In order to project the future incidence cases in China we used a Poisson regression model to fit data from the 5$^{th}$ (start of the incidence declining) to 23$^{rd}$ of February and estimated the decreasing rate per day (z) as:

$$G(t) = G_0 * exp(zt)$$

Where G(t) is the number of new infected at time t and $G_0$ is the initial value at time t=0 (Incidence at day 5 of February). Once the decreasing rate z was estimated and the incidence forecasted from 23 of February onwards, we then calculated the number of infected people coming from China every two weeks period, $A_i(t, t+14)$, as:

$$A_i(t, t+14) = \frac{\sum_{t-14}^{t+14} G(t)}{N} * T(t, t+14)$$

Where N is the total population of China and $T(t, t+14)$ is the number of people travelling from China to Australia in every two weeks period. When calculating the prevalence of infection in China, we started from two weeks before the period travelling in order to include the people that could be infected and in a latent state. In scenarios 2 and 3, we assumed a linear declining distribution in time of travel for university students waiting to enter the country after lifting of the travel ban. A full and partial lifting of the ban was examined. In the partial ban, over 150,000 university students can enter Australia, but the just over 80,000 expected tourists not.

***Epidemic curve in Australia from cases imported from China***

The cases of COVID-19 occurring over time in Australia due to imported cases from China were estimated for each scenario. We used an age specific deterministic model, with 8 mutually exclusive compartments: susceptible (S), Latent traced ($E^t$), Latent untraced ($E^u$), Infectious (I), Isolated (Q), Recovered (R) and dead (D). Each of those compartments is divided in 18 age stratified groups each of 5 years duration, ranging from 0 to 84 years old plus an additional age group of 85+ years. The entire Australian population was considered susceptible. The duration of each model run is 400 days. The initial infected cohort is assumed to be generated from cases arriving from China by air. After arrival of an infected case, it is assumed that, if and when they become symptomatic, they are isolated, and a designated portion of their contacts will also be quarantined. Cases transition between epidemiological compartments in accordance with transition rates determined by their duration of stay in each compartment. Model parameters are shown in Table 1. Further details of the model (diagram and differential equations) are described in the supplementary material. Based on growing evidence of viral loads in asymptomatic cases [12-16], we considered the latent period to be equally infectious as the symptomatic period, however, due to the different length of the two epidemiological states, our assumption results in 41.6 % of the transmissions occurring in the pre-symptomatic state, which is also supported from a recent viral shedding study [17]. The proportion of asymptomatic infections was assumed to be 34.6% based on testing of passengers aboard the Diamond Princess cruise ship [18,19], however we tested in sensitivity analyses the value of 17.9% and 41.6%, which are the range of values estimated for this parameter [20]. The model uses an optimistic assumption that 80% of contacts are identified and quarantined, and 90% of symptomatic cases are isolated after 5 days [21]. Studies show a long, mild prodrome of several days before people feel unwell enough to seek medical attention, which is also considered in the model [21]. We conducted a sensitivity analyses on the proportion of asymptomatic people, R0 and the case detection rate in China. The results are showed in the supplementary material.

**Results**

Figure 1 shows the notified and estimated epidemic in China from the 31 December 2019 to the 23 of February.

Figure 2 shows the modelled epidemic curve fitting the incidence data from 5 to 23 of February and then forecasted until the 4 of April, which is the time we expect the incidence decreasing to almost zero should the current trend in China continue.

We found that following the peak on February 5th and decline of the epidemic in China, the probability that an infected traveller arriving from China under the partial ban scenario (allowing university students only) is low. The complete removal of travel restrictions on 8th of March results in an estimated arrival of 5 cases in the first two weeks and 1 in the following two weeks. However, if we compare a 5 week ban scenario (scenario 2) with the scenario without a travel ban (scenario 1), we estimate that 32, 43 and 36 infected coming every two weeks from the 26 of January would have been averted. Due to a surge of students coming in the first two weeks following the lifting of the ban in the second scenario, an additional 2 more infected are estimated to enter from 8 to 21 of March (Table 2).

In Figure 3 we show the epidemic curve without and with the ban implemented for 5 weeks followed by a full lifting (scenario 1 and 2) and we show a large impact on averting an epidemic in Australia. In both cases, the model reproduces the 15 notified imported cases reported in Australia between the 20 of January and 8 of February. The modelled epidemic in scenario 2, with the full ban, predicts  57 cases in Australia by the 6th of March. The notified cases by 6th of March were 66, however we did not account for imported cases from other countries.

In the epidemic curve for scenario 2, when travel resumes there will be a small surge in cases followed by a decrease and the epidemic can be controlled, with a total of less than 300 cases and about 8 deaths. If the ban was never in place (scenario 1), the epidemic would continue for more than a year resulting in more than 2000 cases and about 400 deaths, in a scenario which considers only localised disease in China.

In each scenario tested, the implementation of the travel ban still reduces the total cases and deaths by about 85%, however in the case of R0 being 3, the travel ban will only delay the epidemic curve pushing the peak about 50 days in time (Figure 2S). The final number of cases and deaths is most sensitive to the R0 assumption, while it is similarly affected by changing the case detection rate (CDR) in China or the proportion of asymptomatic people. Indeed increasing the proportion of asymptomatic people by 2.3 times (from 17.9% to 41.6%), the number of cases increase by 4 times (from a total of 124 to 491 cases with ban on or 975 to over 4000 in the scenario of no ban), while increasing the CDR about 66% (from 30% to 50%) results in 31% decrease in number of cases (from 106 to 76 and from 737 to 461 in the case of travel ban on and off respectively), see Figure 3S and Figure 4S.

**Discussion**

We estimated that the travel ban implemented by Australia on 1 of February, close to the peak of the epidemic in China, has been very effective, reducing the number of cases and deaths from COVID-19 by about 87%. Studies have been published on effectiveness of domestic and international travel restrictions on COVID-19 [22-24]. However, this study is the first one to show the effectiveness of travel bans in Australia, and can inform a phased approach of partial lifting of bans when cases in the source country decline over the course of the pandemic. This allows monitoring of the ongoing situation in China, which may yet see a second wave of the epidemic. The risk of having a person travelling to Australia already infected from China depends from the volume of travel and the infection prevalence in China at that time as well as individual host factors. We used a deterministic compartmental model, so could not consider singular host infectivity and susceptibility, but we acknowledge that these are additional important parameters which could influence the results, and would require an agent based modelling approach to be tested.

When calculating the number of students and tourists arriving from China, we have assumed that the seasonal excess must largely be attributed to the seasonal movement of international students who we estimate represent the large part of the excess movements at this time, which was the most feasible way to account for the normal business and general tourist movements. However the effect of lunar new year on non-student travel is a limitation on this approach.

Our estimate of the true epidemic curve is supported by other studies [10,11,25], and projected case numbers would change with any change in this estimate. Even if the true number of cases in China is 10 or 100 times that reported, only a fraction of the entire population of China has been infected, which leaves a possibility of a subsequent wave of the epidemic. Indeed the fraction of remining susceptibles is still too high to stop transmissions, for an R0 equal to 2.2, about 55% of the population is required to be immune to achieve herd immunity [26]. If cases increase in China, the model can provide estimates of risk based on daily new case numbers.

The pandemic has since surged globally, with new epicentres in Europe and the United States. We do not consider cases coming in from other countries – however, this study illustrates the principle of travel bans and public health impact on epidemic control using China as a case study during a period when the epidemic was largely localised to China. A further limitation is the uncertainty of parameters used, particularly the proportion of asymptomatic cases. Whilst we did not use age weighted assumptions of asymptomatic infection, younger people are more likely to be asymptomatic [20], which would have the effect of increasing undetected transmissions. We have used a conservative estimate, but if the rate is higher than 40%, the outcomes would be worse.

While it has been showed that distancing measures are highly effective [2,27] a systematic review looking at the effectiveness of travel restrictions [28], shows that international travel restriction are effective in delaying an epidemic but may not contain it. We also assumed a very optimistic scenario of 80% of contacts being identified, which may not occur with high case numbers, if a high proportion of asymptomatic transmission is occurring, or if self-quarantine is ineffective. In this study we assumed voluntary home quarantine, which is showed to be about 50% effective in R0 reduction [29], however there could be an increased risk of intra-household transmission infected people to contacts [30], which is not considered in this model.

It has been showed that there could be different transmissions rates per different settings or events, such as super dispersion in a mass gathering [31]. As the model used is a deterministic compartmental model, we could not include different networking or environments. A further limitation of this study is not taking into account diverse transmissions events like super-spreading and lower transmissions. However, the age specific transmission rates allowed us to take into consideration age, which includes heterogeneity in social mixing.

We showed that the ban implemented for travellers from China, when the epidemic was almost at its peak in China, substantially delayed the spread into Australia. There was subsequently evidence of community transmission in Australia and many more cases imported from other countries, but this study provide evidence to support the new travel bans that have been implemented on Italy, Iran and South Korea, in order to delay the epidemic. The model predicted 57 cases by March $6^{th}$ in Australia, which is slightly less than the notified number of 66 on that date, which suggests the model assumptions were reasonable, given we did not account for cases coming in from other countries. Community transmission in Australia in early March is likely linked to imported cases from China, given the fairly long incubation period [32] and less than 3 incubation periods since the first evacuation of Australians from Wuhan on February 3rd. The model fit to observed data was good, also suggesting the epidemic is still possible to contain, if adequate resources are available for thorough contact tracing.

This analyses is a first insight into the effectiveness of travel restrictions for COVID-19 outbreak, supports the effectiveness of the Australian response, informs gradual lifting of the bans or placing of new bans on other countries, and could inform other countries in reducing the burden of importations and resulting domestic transmission of COVID-19.

***Table 1: Parameters used in the model***

| **Parameter** | **Value** | **Source** |
|---|---|---|
| Basic reproduction number | 2.2, sensitivity analyses as 1.8 and 3 | [1] |
| Infectious period | 12.2 days of which 5.2 asymptomatic and 7 | [1] |

| | | |
|---|---|---|
| | symptomatic | |
| Time to isolation once symptomatic | 5 days | [21] |
| Effectiveness of home quarantine | 50% reduction in the R0 | [29] |
| Duration of home quarantine | 14 days | Australian recommendation |
| Duration of hospital isolation | 20 days | |
| Proportion of asymptomatic or very mild infectious | 34.6% (17.9% and 41.6% used in sensitivity analyses) | [18-20] |
| Proportion of contacts identified for home quarantine | 80% | [33] |
| Chinese detection rate | 10%, with sensitivity analyses as 30% and 50% | [10] |
| Proportion of symptomatic people that get isolated after 5 days | 90% | [33] |
| Age-specific case fatality rate (%) for the 18 age groups | 0, 0, 0.2, 0.2, 0.2, 0.2, 0.2, 0.2, 0.4, 0.4, 1.3, 1.3, 3.6, 3.6, 8, 8, 14.8, 14.8 | [34] |

***Table 2: Imported cases in Australia from China under no, partial and full travel bans per each two weeks period considering disease in China only***

| **Time travelling** | **Infected entering Australia without ban (scenario 1)** | **Infected entering Australia with ban from 1 February to 7 of March followed by full lifting of ban (scenario 2)** | **Infected entering Australia with following a ban from 1 February to 7 of March followed by partial lifting of ban (scenario 3)** |
|---|---|---|---|
| 26 January to 8 February | 39 | 7 | 7 |
| 9 to 22 February | 43 | 0 | 0 |
| 23 February to 7 March | 36 | 0 | 0 |
| 8 to 21 March | 3 | 5 | 0 |
| 22 March to 4 April | 1 | 1 | 0 |

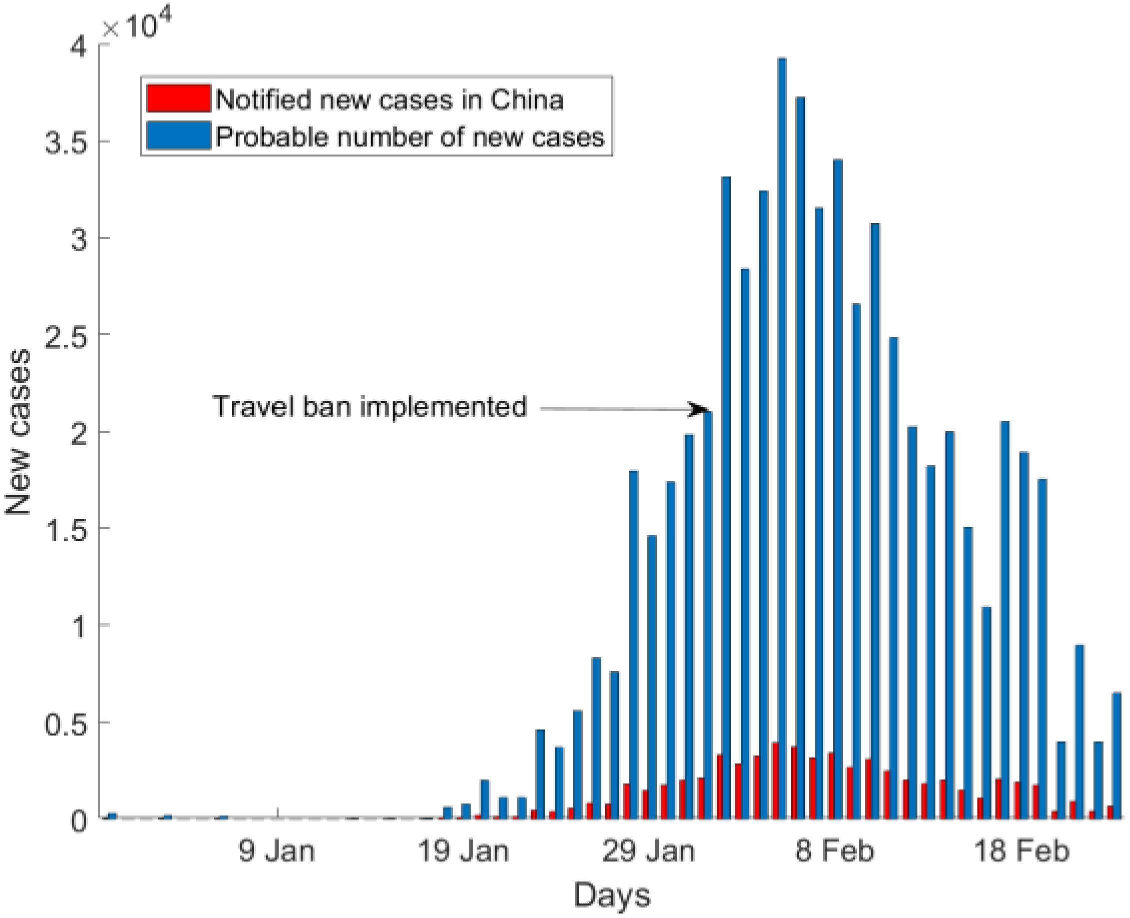

***Figure 1: the estimated true epidemic curve (blue) compared to the reported epidemic curve in China (red)*** [10]

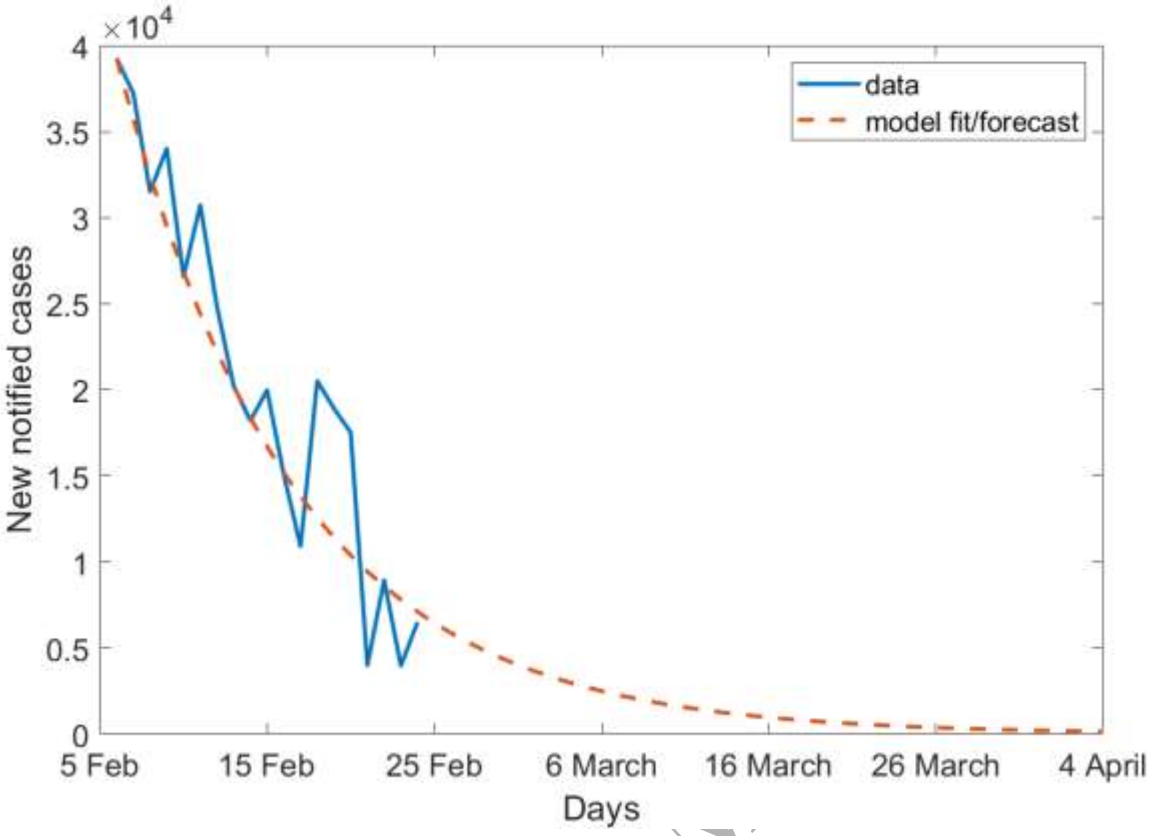

***Figure 2: Estimated Incidence data in China (blue) and model fit to the data and forecasting future daily incidence (red)***

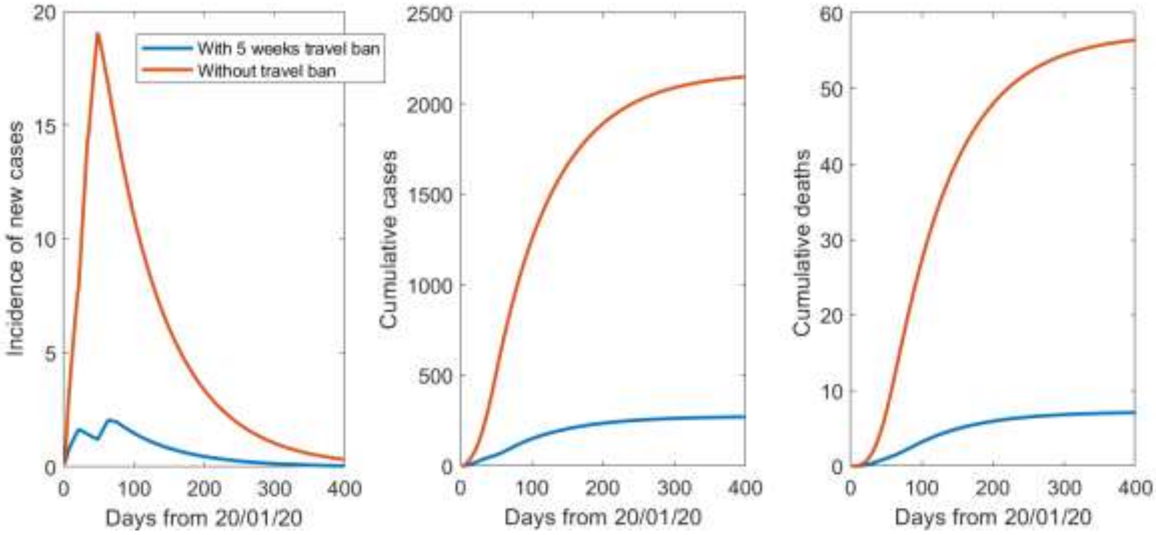

***Figure 3: Daily incidence, cumulative number of cases and the cumulative number of deaths from the 20 of January onward for 400 days with and without a travel ban***
